# Genome-Wide Identification of the *PR-1* Gene Family in *Pyrus betulaefolia* Bunge and Its Expression Analysis Under Fire Blight Stress

**DOI:** 10.3390/ijms26115074

**Published:** 2025-05-24

**Authors:** Abudusufuer Wufuerjiang, Jingyi Sai, Yue Wen, Lei Wang, Chen Chen, Xu Li, Jia Tian

**Affiliations:** 1 College of Horticulture, Xinjiang Agricultural University, Urumqi 830052, China; sufuer715@163.com (A.W.); cyberone12@163.com (J.S.); wenyue900701@163.com (Y.W.); ewlei@163.com (L.W.); m18434365917@163.com (C.C.); 2 Emin County of Agriculture and Rural Development, Tacheng 834600, China; 15909016219@163.com

**Keywords:** *Pyrus betulaefolia* Bunge, PR-1, fire blight, plant defense, bioinformatics analysis

## Abstract

Fire blight, caused by *Erwinia amylovora*, is a devastating bacterial disease threatening apple, pear, and other Rosaceae species. In our prior study, transcriptome analysis identified a fire blight-resistant variety, Duli (*Pyrus betulifolia* Bunge), and highlighted the PR1 gene as a key resistance factor. Using Duli’s genomic data, we systematically identified and characterized the *Pb-PR-1* gene family through bioinformatics analysis. A total of 31 *Pb-PR-1* genes were found, encoding proteins of 123–341 amino acids. Phylogenetic analysis grouped these genes into four subfamilies, with 27 genes distributed across seven chromosomes, all contain a conserved CAP superfamily domain. Their promoter regions were enriched in hormone and stress-responsive elements. After inoculation with *E. amylovora*, susceptible Duli showed lesion development by day 2, with rapid disease progression, while resistant plants exhibited slower disease advancement and smaller lesions. Enzyme activity assays revealed that in resistant plants, PPO (polyphenol oxidase) and CAT (catalase) activities peaked on day 6, showing a 2.4-fold and 3.81-fold increase compared to susceptible Duli. At the same time, MDA (malondialdehyde) content decreased by 16.6%. The activities of SOD (superoxide dismutase) and PAL (phenylalanine ammonia-lyase) peaked on day 4, with increments of 34.32% and 47.1% over susceptible Duli. qRT-PCR showed significant differences in *Pb-PR-1* gene family expression between resistant and susceptible plants post-inoculation. Notably, *Pb-PR-1-11*, *Pb-PR-1-21*, and *Pb-PR-1-26* expression increased with infection duration, aligning with PPO and CAT activity trends. Other genes showed high early infection expression but declined by day 6. *Pb-PR-1-3*, *Pb-PR-1-6*, *Pb-PR-1-8*, *Pb-PR-1-16*, and *Pb-PR-1-30* were upregulated 13.17-fold on average by day 2. In summary, the *Pb-PR-1* family exhibited elevated expression during early infection and enhanced defense-related enzyme activities, improving plant resistance. This study provides a foundation for understanding the *PR-1* family’s role in Duli and advancing fire blight resistance in Pyrus species.

## 1. Introduction

Throughout the extended evolutionary process, plants have developed a sophisticated immune system to effectively combat a diverse array of pathogens. This immune response is primarily anchored in two key defense mechanisms: Pathogen-Associated Molecular Pattern (PAMP)-triggered immunity (PTI) and effector-triggered immunity (ETI) [1]. PTI serves as the initial line of defense, activated when plants identify Pathogen-Associated Patterns (PAMPs) through pattern recognition receptors (PRRs). Conversely, ETI acts as a second line of defense, initiated by the recognition of pathogen-secreted effectors by plant resistance proteins (R proteins) [2]. Within these immune responses, pathogenesis-related proteins (PR proteins) play a crucial role in mediating and enhancing plant defense mechanisms.

PR proteins are induced in plants in response to pathogen infection or other stressors. These proteins are classified into 17 distinct families, designated PR1 to PR17 [3]. PR1 proteins are recognized for their antimicrobial effects, whereas PR5, PR12, PR13, and PR14 exhibit fungicidal properties, and PR10 is capable of degrading viral RNA [4,5]. The *PR-1* gene, in particular, has received considerable attention due to its role in conferring resistance against fungi, oomycetes, and bacteria [6]. Initially identified in tobacco plants infected with Tobacco Mosaic Virus (TMV), high expression levels of the *PR-1* gene indicated its induction during pathogen infection and its involvement in disease resistance [7]. In kiwifruit, the *AePR-1* gene exhibits significant expression under the induction of *Pseudomonas syringae pv*. actinidiae and hormones, and its overexpression enhances resistance to bacterial canker [8]. Overexpression of the pepper basic *PR-1* gene (*CAB-PR1*) in tobacco has been shown to increase resistance to *Phytophthora nicotianae* and *Ralstonia solanacearum* [9]. These findings suggest that overexpression of *PR-1* genes can bolster plant resistance to pathogens, underscoring the significance of *PR-1* genes in plant immune defense [10]. A conserved domain is a region within a protein sequence that has remained relatively unchanged over the course of evolution. This conservation implies that the domain is functionally important [11]. PR-1 proteins belong to the CAP protein superfamily, which includes a conserved CAP domain. The antimicrobial activity of the CAP domain is ascribed to two primary factors: the presence of a cavity protein-binding motif (CBM) that binds to and sequesters sterols from pathogen membranes, and the CAP-derived peptide (CAPE) derived from the last 11 residues of the CAP domain, which initiates defense responses via an independent signaling pathway [12].

The initial response of the host to pathogen attack is characterized by the hypersensitive response, which triggers the production of reactive oxygen species (ROS), primarily hydrogen peroxide (H_2_O_2_) and superoxide anion (O_2_^−^) [13,14]. These ROS play a critical role in activating plant defense mechanisms, including programmed cell death, and function as sophisticated secondary messengers, inducing various genes and signaling cascades associated with plant defense [15,16]. However, excessive or improper production of ROS can adversely affect cellular components, leading to damage to DNA, proteins, and lipids, ultimately resulting in cell death [17]. To mitigate these detrimental effects, ROS-scavenging enzymes such as superoxide dismutase (SOD), peroxidase (POD), and catalase (CAT) are essential in maintaining ROS homeostasis and aiding plants in resisting pathogen-induced stress [18,19]. For instance, research on the resistance of leaves from four early-maturing pear varieties to black spot disease revealed that following pathogen infection, the activities of POD, SOD, and CAT significantly increased in the leaves of Cuiguan pear and Sucui No. 1 pear [20]. In studies examining the defense responses of two apple varieties, one susceptible and one resistant to fire blight, it was found that the resistant variety exhibited higher enzymatic activity, particularly phenylalanine ammonia-lyase (PAL), which effectively inhibited pathogen growth [21]. Additionally, *Pyrus* ussuriensis has demonstrated a level of resistance against pear fire blight; after inoculation with the fire blight pathogen, the activities of CAT, POD, and PAL in this variety were significantly elevated compared to the susceptible variant [22]. Further investigations revealed that resistance mediated by the *PR-1* gene closely correlates with the regulation of defense enzymes. In research focusing on rice sheath blight, it was observed that post-infection, the expression levels of *PR* genes and the activities of SOD, CAT, and PAL in the leaves and leaf sheaths of two rice varieties increased. The resistant variety displayed a heightened defense response, characterized by elevated PR protein levels and defense enzyme activity [23]. In studies concerning tomato resistance to *Fusarium oxysporum f. sp*, a positive correlation was identified between the increased activity of SOD and the high expression of the *PR-1* gene [24]. SOD plays a crucial role in scavenging ROS produced during pathogen infection, thereby safeguarding plant cells [24]. These findings underscore the multifaceted role of *PR-1* in orchestrating the enzyme-defense network, highlighting its significance in plant defense mechanisms.

Fire blight, caused by the bacterium *Erwinia amylovora*, stands as one of the most destructive diseases affecting Rosaceae plants [25]. Initially identified in 1780 in New York State and the Hudson Highlands of the United States [26], fire blight was first documented in Yili Kazakh Autonomous Prefecture, Xinjiang (China) in 2016 and subsequently spread to Korla in 2017 [27]. Data indicate that the local pear industry has experienced a production decline exceeding 200,000 tons as a result of this disease, leading to substantial economic losses [28,29]. While Duli is a widely adopted rootstock for cultivated pear varieties in northern China due to its notable stress adaptability, its resistance to fire blight is relatively limited [30,31]. Consequently, the cultivation of disease-resistant rootstock varieties has emerged as the most effective strategy for controlling fire blight, especially in light of the significant threats posed to scion varieties. Current management strategies for fire blight in scion varieties include comprehensive measures such as pruning, chemical applications, and biological controls. However, effective prevention and control methods for rootstocks remain lacking. In previous research conducted by our team, RNA-seq technology was utilized for transcriptome analysis on both resistant and susceptible Duli germplasms under fire blight stress, leading to the identification of numerous differentially expressed genes [32]. Notably, the *PR-1* gene exhibited significant variations across different infection stages of resistant and susceptible materials.

Building on this foundational research, the present study employs publicly accessible Duli genomic data to conduct a genome-wide identification and analysis of the *PR-1* gene family within Duli, a candidate gene associated with resistance. This investigation will assess the levels of defense-related enzymes at various time points post-fire blight stress, alongside the differential expression of *PR-1* gene family members. Findings from this study aim to deepen our understanding of the functions and molecular regulatory mechanisms of the *PR-1* gene family in response to fire blight stress, thereby providing a crucial theoretical framework for the further genetic enhancement of fire blight resistance in *Pyrus* species.

## 2. Results

### 2.1. Physicochemical Property Analysis of the Pb-PR-1 Gene Family Proteins

Utilizing genomic data from Duli, a total of 31 *PR-1* genes were identified and sequentially assigned designations from *Pb-PR-1-1* to *Pb-PR-1-31* (Table 1). An analysis of the fundamental characteristics of the *Pb-PR-1* gene family proteins revealed that the number of encoded amino acids varies from 123 to 341 (with Pb-PR-1-2 being the largest). The relative molecular mass of these proteins ranges from 13,268.7 Da to 37,556.87 Da, and the isoelectric points vary between 4.67 and 9.85. The instability index for these proteins shows a range from 21.85 to 43.45, while the grand average of hydropathicity (GRAVY) values falls between 57.48 and 82.88. Importantly, the overall average hydrophilicity of the identified family proteins is negative, indicating that they are primarily hydrophilic proteins. Predictions regarding subcellular localization suggest that two of the PR-1 proteins are localized to the nucleus, while the remaining twenty-nine are found in the vacuole (the presented subcellular localization results are only potentially predicted outcomes). These findings emphasize the variability in molecular weight and hydrophilicity among different members of the *Pb-PR-1* gene family, suggesting that these proteins may play diverse roles in cellular functions. For example, some PR-1 proteins may function in signaling pathways to initiate defense responses, while others could be involved in the production of reactive oxygen species (ROS) or the modification of cell wall components to enhance pathogen resistance [33,34].

### 2.2. Chromosomal Localization of Pb-PR-1 Gene Family

**Figure 1:**
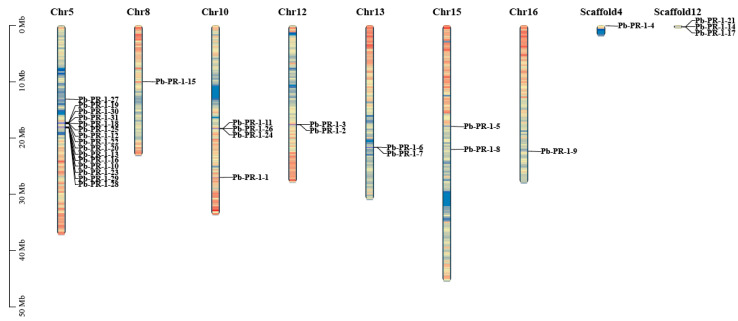
Chromosomal location of *Pb-PR-1* gene family.

In order to elucidate the chromosomal distribution of *PR-1* gene family in Duli, a total of 31 genes within the family were subjected to chromosomal mapping utilizing TBtools and genome annotation files (Figure 1). The analysis demonstrated that 27 of these genes are dispersed across chromosomes 5, 8, 10, 12, 13, 15, and 16, indicating an uneven distribution pattern. Notably, chromosome 5 harbors the highest concentration, comprising fifteen genes (*Pb-PR-1-10*, *-12*, *-13*, *-16*, *-18*, *-19*, *-20*, *-22*, *-23*, *-25*, *-27*, *-28*, *-29*, *-30*, *-31*), with two of these genes arranged in tandem. This arrangement suggests that, upon induction and activation, these genes may be expressed rapidly in clusters. Furthermore, one gene was observed on each of chromosomes 8 and 16, respectively, *Pb-PR-1-15* and *Pb-PR-1-19*.

### 2.3. Gene Duplication and Collinearity Analysis of PbPR-1 Gene Family Members in Duli and Arabidopsis

**Figure 2:**
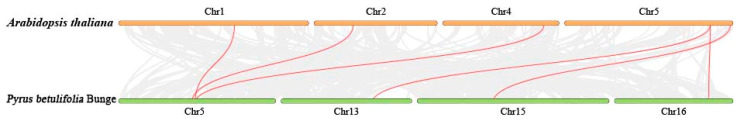
Analysis of covariance between Duli and *Arabidopsis thaliana*. Note: The red lines indicate genes within these two species that exhibit collinearity relationships, while the gray color denotes genes that do not show any covariant relationships.

A comparative analysis with the *Arabidopsis* reference genome revealed that six out of the thirty-one *Pb-PR-1* gene family members exhibit collinearity, located on chromosomes 5, 13, 15, and 16. This finding provides a valuable reference for the functional study of *Pb-PR-1* genes (Figure 2).

### 2.4. Phylogenetic Tree Analysis of Pb-PR-1 Gene Family Members

**Figure 3:**
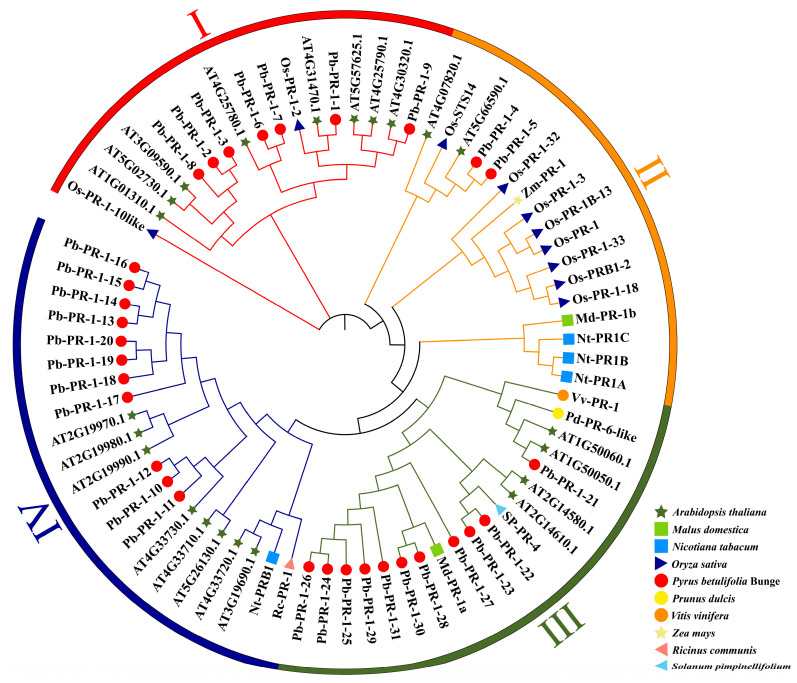
Phylogenetic tree of Pb-PR-1 gene family proteins and other plant proteins. Note: The various colors displayed on the exterior of the tree signify their respective groups. Specifically, orange, yellow, dark green, and dark blue represent Group 1, Group 2, Group 3, and Group 4, respectively. At-*Arabidopsis thaliana*; Md-*Malus domestica*; Nt-*Nicotiana tabacum*; Os-*Oryza sativa*; Pb-*Pyrus betulifolia* Bunge; Pd-*Prunus dulcis*; Vv-*Vitis vinifera*; Zm-*Zea mays*; Rc-*Ricinus communis*; Sp-*Solanum pimpinellifolium*.

The examination of proteins within the same family reveals frequent functional similarities, which are manifested through the conservation of their encoded amino acid sequences. To clarify the phylogenetic relationships among *PR-1* gene family proteins, BLAST (https://blast.ncbi.nlm.nih.gov/Blast.cgi, accessed on 20 September 2024) alignments were conducted using PR-1 protein sequences as queries in various databases. The analysis incorporated Duli and *Arabidopsis* PR-1 proteins, along with their homologous counterparts, to construct a comprehensive phylogenetic tree (Figure 3). The analysis revealed that the 31 Pb-PR-1 proteins, in addition to PR-1 proteins from species such as grape, tobacco, rice, maize, tomato, and plum, exhibited distinct clustering patterns within the phylogenetic tree. As illustrated in Figure 3, these proteins can be categorized into four subgroups based on their clustering behaviors. Notably, the number of PR-1 family members varies across subgroups: Subgroup 1 has seven members, Subgroup 2 has two, and Subgroups 3 and 4 each contain eleven. In Subgroup 2, Pb-PR-1-4 and Pb-PR-1-5 are more distantly related to other family members, suggesting early divergence during evolution. Conversely, members in Subgroups 1, 3, and 4 exhibit close relationships and distinct clustering. The phylogenetic tree indicates that Pb-PR-1-1 is closely related to Os-PR-1-2, Pb-PR-1-4 to Os-STS14, Pb-PR-1-22 and Pb-PR-1-23 to SP-PR-4, and Pb-PR-1-28 and Pb-PR-1-30 to Md-PR-1a, all within the same clade. Os-PR-1-2 and Os-STS14 enhance rice blast resistance [35], while SP-PR-4 boosts tomato bacterial wilt resistance [36], implying similar functions for these members. Functions of other members in the tree remain unverified. Overall, Pb-PR-1 shows closer relationships with PR-1 proteins from other species.

### 2.5. Analysis of Gene Structure and Conserved Motifs in the Pb-PR-1 Gene Family

**Figure 4:**
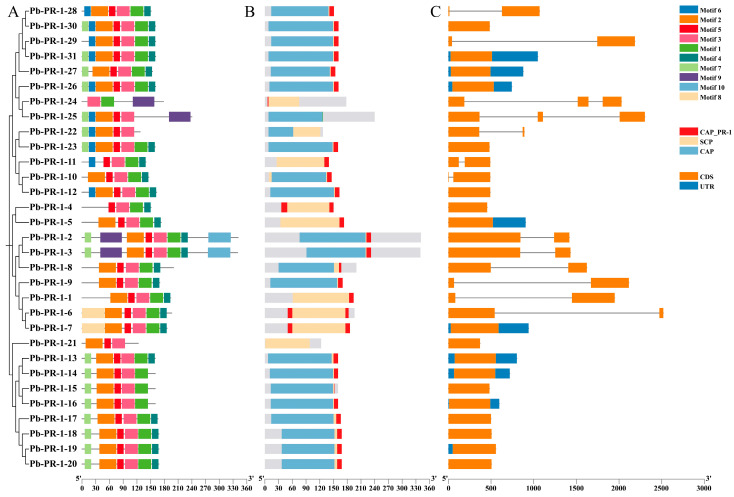
The analysis focuses on the conserved motifs, gene structure and structural domains of the *Pb-PR-1* gene family. Note: (**A**) The conserved motifs are displayed with different colors corresponding to distinct motifs, maximum of 10 motifs were searched with motif length between 10 and 50 residues. (**B**) An overview of the conserved structural domains is provided as well. (**C**) The cluster analysis illustrates the gene structure, where the blue box denotes the UTR, the orange box indicates the CDS, and the line represents the introns.

Further examination of the 31 protein sequences within the Pb-PR-1 gene family, utilizing MEME7.0 and TBtools II software, identified a total of 10 conserved motifs (Figure 4A). Notably, five motifs (motif 1 to motif 5) were consistently located across most family members, indicating a high level of conservation. Pb-PR-1-2 and Pb-PR-1-3 exhibited the greatest number of motifs, with eight each, while Pb-PR-1-21 and Pb-PR-1-24 displayed the fewest, at three. Additionally, motif 10 was exclusively found in Pb-PR-1-2 and Pb-PR-1-3, and motifs 9 and 10 were observed in only 3-5 of the family members. The arrangement of these conserved motifs among the 31 Pb-PR-1 protein members underscores the significant conservation within the *Pb-PR-1* gene family. Prediction analyses conducted via NCBI-CDD confirmed that 19 out of the 31 selected member proteins harbored a CAP domain, spanning between residues 20-185 (Figure 4B). Notably, Pb-PR-1-2 and Pb-PR-1-3 exhibited a CAP domain range of 95-230, Pb-PR-1-21 has the SCP domain. All other family members contained the CAP_PR-1 domain, reinforcing the conclusion that all members are part of the CAP superfamily. A comprehensive analysis of the *Pb-PR-1* gene family members was conducted using TBtools to elucidate their gene structure and conserved motifs. The results indicated that, with the exception of *Pb-PR-1-5*, *Pb-PR-1-7*, *Pb-PR-1-13*, *Pb-PR-1-14*, *Pb-PR-1-16*, *Pb-PR-1-19*, *Pb-PR-1-26*, *Pb-PR-1-27*, and *Pb-PR-1-31*, all other family members lack untranslated regions (UTRs), which may have implications for mRNA transport, translational regulation, and overall gene annotation (Figure 4C).

### 2.6. Analysis of Cis-Acting Elements in the Promoter of Pb-PR-1 Gene Family Members

**Figure 5:**
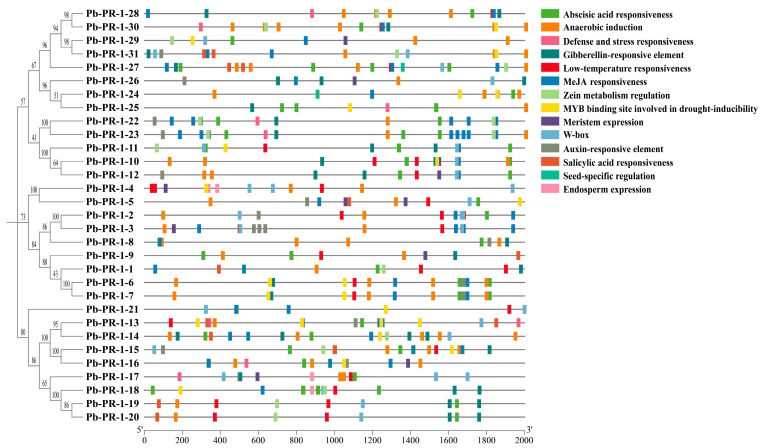
Cis-acting elements predicted in 2000 bp sequence upstream of the *Pb-PR-1* gene. Different colored squares represent various types of cis-acting elements.

This study aims to elucidate the functions of the *Pb-PR-1* gene family and identify conserved cis-acting elements within the promoter regions of *Pb-PR-1* genes. A comprehensive analysis was conducted utilizing the PlantCARE (http://www.bioinformatics.psb.ugent.be/webtools/plantcare/html/, accessed on 20 September 2024) database, which uncovered three primary categories of cis-acting elements: biotic and abiotic stress responses, plant hormone interactions, and elements related to plant growth and development (Figure 5). Overall, a total of 14 distinct types of elements were predicted (abscisic acid responsiveness, anaerobic induction, defense and stress responsiveness, gibberellin-responsive element, low-temperature responsiveness, MeJA-responsiveness, zein metabolism regulation, MYB binding site involved in drought-inducibility, meristem expression, W-box, auxin-responsive element, salicylic acid responsiveness, seed-specific regulation, endosperm expression). The promoter regions of Pb-PR-1 family members contain various elements related to plant growth, development, and hormone responses. Specifically, these include 15 auxin response elements (AuxREs), 47 abscisic acid response elements (ABREs), and 55 jasmonic acid response elements (TGACG-motif, CGTCA-motif). Methyl jasmonate (MeJA), an important hormone in plants, plays a crucial role in numerous physiological processes. Additionally, 13 salicylic acid (SA) response elements were identified, underscoring the key role of hormones in plant growth and development. Furthermore, 68 anaerobic induction elements (AREs), 28 W-boxes (WRKY boxes) linked to wound and pathogen responses, and 23 MYB stress-response elements were detected across multiple Pb-PR-1 family members, accounting for 33.80% of the total elements. Moreover, the promoter regions of *Pb-PR-1* genes also exhibited defense and stress response elements, low-temperature response elements, and MYB binding sites associated with drought induction, further emphasizing the involvement of *Pb-PR-1* genes in plant defense physiology.

### 2.7. Secondary Structure Prediction and 3D Modeling of the Pb-PR-1 Gene Family Proteins

**Figure 6:**
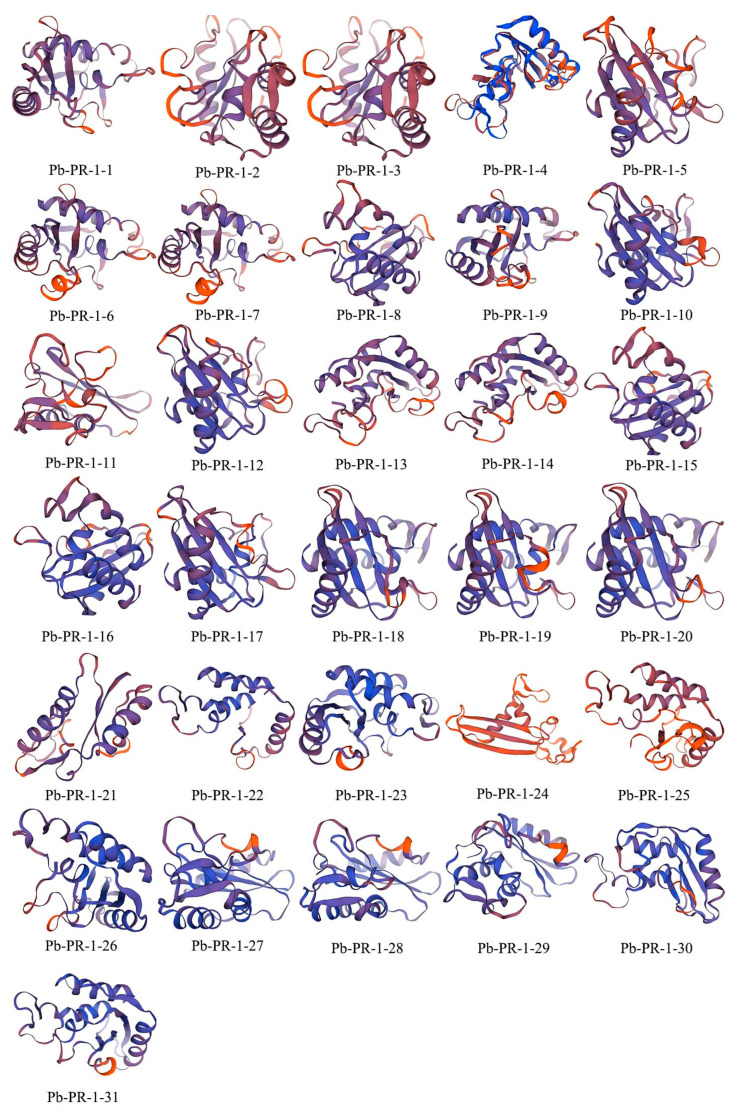
Models of the tertiary structure of the Pb-PR-1 gene family proteins.

The computational analysis of the secondary structure of Pb-PR-1 gene family proteins revealed considerable variation in the content of α-helices, β-turns, random coils, and extended strands. Specifically, the α-helices content ranged from 28 to 40%, while β-turns constituted 2–7%. Random coils comprised a substantial 39–53%, and extended strands accounted for 10–23%. The relative scarcity of β-turns suggests their potential role in auxiliary modifications, as illustrated in Table S2. Overall, α-helices and random coils were the predominant structural elements across the protein family. The three-dimensional conformation modeling of the Pb-PR-1 proteins indicated significant structural similarities among Pb-PR-1-2, -3, -6, -7, -13, -14, -18, -19, -20, -27 and Pb-PR-1-28 (Figure 6). In contrast, Pb-PR-1-11 and Pb-PR-1-24 displayed distinct structural characteristics, characterized by a dense arrangement of helical elements, highlighting both conservation and divergence within the family.

### 2.8. Phenotypic Analysis of Resistant and Susceptible Duli After Inoculation

**Figure 7:**
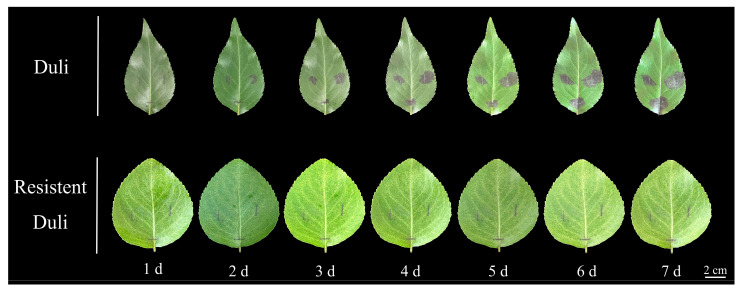
The susceptibility of Duli and resistant Duli leaves one to seven days after inoculation with fire blight.

Following inoculation with a bacterial suspension of fire blight on detached leaves of both resistant and susceptible Duli, distinct variations in disease symptomatology were observed (Figure 7). Preliminary trials indicate no direct relationship between morphology and disease resistance. The lesions exhibited a progressive expansion from the points of injury, characterized by a dark brown pigmentation. Notably, some leaves displayed the formation of dark black mucus, with lesion lengths increasing correspondingly with the days post-inoculation. In susceptible Duli leaves, lesions began to spread as early as the second day post-inoculation. Over time, the affected area notably increased, with the size of the lesions in susceptible Duli being significantly greater than those observed in resistant specimens. The progression of disease in the susceptible variety of Duli showed a deceleration by the sixth day. Conversely, the disease-resistant Duli was not susceptible to the disease 1–7 d after inoculation, and the brown phenomenon appeared in the incised site at the later stage after inoculation. The rate of disease progression was markedly slower in this group compared to the susceptible Duli, maintaining a consistent disease status. It is essential to emphasize that the lesion area in susceptible Duli was substantially larger than that in their resistant counterparts, demonstrating a positive correlation between the length of lesions and the duration of inoculation.

### 2.9. Enzymatic Activity and MDA Content Analysis of Resistant and Susceptible Duli After Inoculation

**Figure 8:**
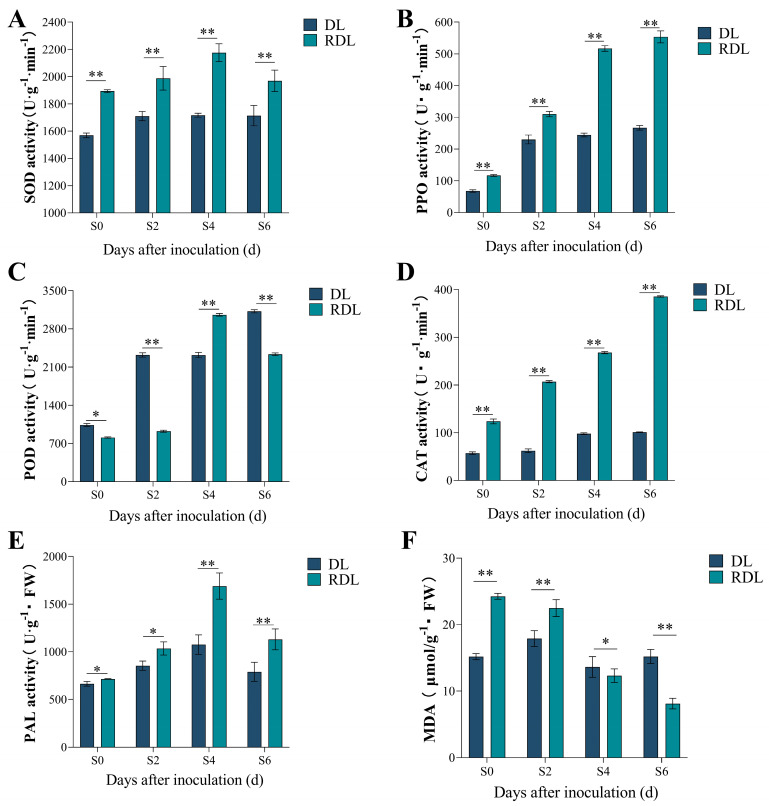
Changes in defense enzyme activity after inoculation. Note: In the figure, (**A**) The Changes in SOD activity of the resistant Duli and Duli after fire blight treatment; (**B**) The Changes in PPO activity of the resistant Duli and Duli after fire blight treatment; (**C**) The Changes in POD activity of the resistant Duli and Duli after fire blight treatment; (**D**) The Changes in CAT activity of the resistant Duli and Duli after fire blight treatment; (**E**) The Changes in PAL activity of the resistant Duli and Duli after fire blight treatment; (**F**) The Changes in MDA content of the resistant Duli and Duli after fire blight treatment; * represents *p* < 0.05; ** represents *p* < 0.01. “DL” represents Duli; “RDL” represents resistant Duli.

The analysis of leaf-related enzyme activities and malondialdehyde (MDA) content following pathogen infection (Figure 8) reveals significant insights into the physiological responses of Duli. In the resistant variants, the activities of SOD and PAL exhibited a pattern of initial increase (from the Sck stage to S4) followed by a decrease at S6, with peak values recorded at the S4 stage. Specifically, the levels of SOD and PAL in the resistant Duli reached 2176.009 U·g^−1^·min^−1^ and 1688.334 U·g^−1^·FW, respectively, both of which were significantly elevated compared to those observed in susceptible Duli (Figure 8A,E). In contrast, the enzyme activities for SOD and PPO in the susceptible variants remained stable throughout the S2 to S6 timeframe (Figure 8A,B). This stability suggests that the susceptible plants experienced considerable stress from fire blight, leading to detrimental effects on cellular integrity and an inability to accumulate resistance-related compounds.

Additionally, the activity of CAT in the resistant Duli showed a positive correlation with the duration post-inoculation, peaking on the 6th day at 385.44 U·g^−1^·min^−1^, 73.77% higher than that of the susceptible variants (Figure 8D). This indicates that the upregulation of CAT enzyme activity post-infection plays a crucial role in the degradation of H_2_O_2_, thereby bolstering the plant’s defensive mechanisms. The MDA content in susceptible Duli demonstrated an initial increase, followed by a subsequent decrease and then a rise again. Conversely, the resistant variants displayed a continuous decrease in MDA levels as the duration of inoculation extended, illustrating an inverse relationship. Notably, during the early infection stages (Sck to S2), the MDA content in resistant Duli was 26.18% and 39.38% higher than in susceptible plants (Figure 8F). These findings underscore that germplasms exhibiting disease-resistant traits can promptly adapt and respond to fire blight invasions by effectively reducing MDA levels, which consequently enhances their resistance capabilities.

### 2.10. Differential Expression Analysis of Pb-PR-1 Gene Family in Response to Fire Blight Infection

**Figure 9:**
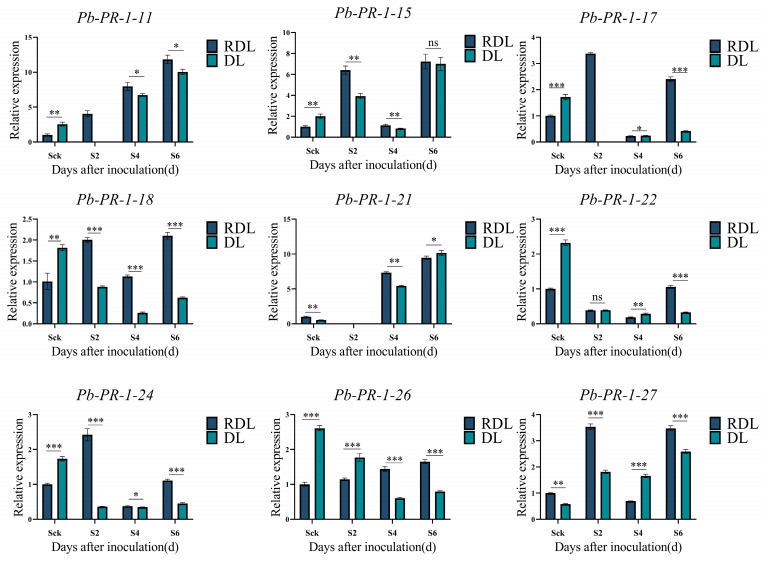
Relative expression of the *Pb-PR-1* gene family after inoculation with fire blight. Note: In the accompanying figure, the symbols denote statistical significance, where * indicates *p* < 0.05, ** signifies *p* < 0.01, and *** denotes *p* < 0.001. The label “ns” refers to no significant difference. Additionally, “DL” represents Duli, categorized by its susceptibility, while “RDL” denotes the resistant Duli.

To elucidate the regulatory dynamics of the *Pb-PR-1* gene family in response to fire blight infection, we conducted an analysis of the expression profiles of *PR-1* genes in both resistant and susceptible Duli at 2, 4, and 6 days post-infection (dpi) utilizing RT-qPCR, with non-infected controls (Sck) serving as a reference (Figure 9 and Figure S1). The expression of *Pb-PR-1-3*, *Pb-PR-1-6*, *Pb-PR-1-8*, *Pb-PR-1-16*, and *Pb-PR-1-30* exhibited significant upregulation in resistant varieties (S2) by 12.2, 10.9,18.38, 13.67, and 10.7-fold, respectively. In contrast, susceptible Duli demonstrated an average upregulation ranging from 3- to 5-fold. Notably, *Pb-PR-1-4* and *Pb-PR-1-21* were expressed at the second dpi in both resistant and susceptible varieties, while *Pb-PR-1-2*, *Pb-PR-1-11*, and *Pb-PR-1-17* were absent in susceptible varieties at the second and fourth dpi, with expression levels showing an increase at later stages. In resistant Duli, the expression of *Pb-PR-1-11*, *Pb-PR-1-21*, and *Pb-PR-1-26* exhibited a positive correlation with the duration of infection (Figure 9), whereas *Pb-PR-1-17*, *Pb-PR-1-19*, and *Pb-PR-1-22* demonstrated a decline over time, but the expression levels of most family members decreased with the infection time after the S2 period (Figure 9). A subset of genes, including *Pb-PR-1-15*, *Pb-PR-1-17*, *Pb-PR-1-18*, *Pb-PR-1-22*, *Pb-PR-1-24*, and *Pb-PR-1-27*, exhibited a biphasic expression pattern characterized by an initial increase, subsequent decrease, and later resurgence (Figure S1). Overall, all *Pb-PR-1* genes showed varying degrees of upregulation following infection, with a discernible trend of increased expression during the early phase (S2), a reduction during the middle phase (S4), and a resurgence in expression during the later phase (S6). Furthermore, the expression levels of the *Pb-PR-1* gene family in resistant Duli significantly exceeded those observed in susceptible individuals, suggesting a potential role for *Pb-PR-1* genes in mediating resistance to fire blight.

In conjunction with the analysis of enzyme activities, we observed that in resistant Duli at the S6 stage post-inoculation, the expression levels of genes *Pb-PR-1-11*, *Pb-PR-1-21*, and *Pb-PR-1-26* peaked. Concurrently, the activities of CAT and PPO also reached their maximum at this stage. During the early phase of fire blight infection, the expression levels of *Pb-PR-1-1*, *Pb-PR-1-3*, *Pb-PR-1-7*, *Pb-PR-1-9*, *Pb-PR-1-15*, *Pb-PR-1-16*, *Pb-PR-1-18*, *Pb-PR-1-26*, *Pb-PR-1-29*, and *Pb-PR-1-31* were elevated compared to those in susceptible Duli, correlating with heightened activities of SOD, POD, and PAL at the same stage (Figure 8). These findings underscore the notion that the heightened expression of *PR-1* gene family at various stages enhances the activity of antioxidant enzymes, thereby bolstering the resistance of resistant Duli to fire blight.

## 3. Discussion

PR proteins are critical signaling molecules that plants produce in response to biotic and abiotic stresses. The expression of PR proteins is induced by pathogen infection, thereby enhancing the plant’s resistance mechanisms [2]. Variations in the *PR-1* gene family across species have been documented, with a total of 31 *PR-1* family members identified in Duli, a number that differentiates it from other species. For reference, there are 13 *PR-1* members in tomato [37], 24 in soybean [38], 38 in rice [35], 10 in mango [39], and 20 in barley [40]. This variability in family member numbers is likely attributable to specific gene amplification events experienced by the *Pb-PR-1* family across different species [41]. The observed changes in the number of *PR-1* genes can be linked to gene duplication events or variations in genome size, both of which underscore the functional diversity and evolutionary trajectory of *PR-1* genes in various species [42]. While much of the gene family has remained evolutionarily conserved, certain genes within the family have exhibited accelerated evolutionary rates due to functional constraints, potentially affecting the length of their amino acid residues [41,42].

Our analysis of the Pb-PR-1 family revealed that the amino acid residue lengths of Pb-PR-1-2, Pb-PR-1-3, and Pb-PR-1-25 range from 200 to 341. Notably, PR proteins are generally characterized as low molecular weight proteins, approximately 6–43 kDa, with typical amino acid residue lengths ranging between 120 and 196 residues [43]. The predicted amino acid lengths in this study exceed the standard range by 0.5 to 1 times, suggesting that variations among family members may arise from differential molecular evolutionary rates [44]. PI analysis of the *PR-1* gene family identified 11 basic proteins localized intracellularly and 20 acidic proteins situated extracellularly. The distribution and pI characteristics of these proteins are closely related to the functional diversity within this gene family, indicating that the acidic and basic properties of PR-1 proteins influence their cellular localization and, consequently, their roles in plant defense responses [45]. Phylogenetic analysis reveals that member of the *Pb-PR-1* gene family cluster with PR-1 proteins from various plant species, illustrating their evolutionary connections. Notably, Pb-PR-1-4 and Pb-PR-1-5 exhibits a more distant phylogenetic relationship with other family members, suggesting its potential involvement in several independent evolutionary events [40]. Conversely, Pb-PR-1-22 and Pb-PR-1-23 to shares a closer phylogenetic relationship with SP-PR-4, mirroring the relationship between the disease-resistant tomato SlPR1b and its homologous proteins [36]. This similarity in evolutionary relationships leads to the hypothesis that PR-1 proteins in Duli may also play roles in conferring disease resistance. Furthermore, among the 15 family members located on chromosome 5 and on chromosome 10, specific genes such as *Pb-PR-1-17*, *Pb-PR-1-20*, and *Pb-PR-1-26* are tandemly arranged, despite not being in the same branch of the phylogenetic tree. This tandem arrangement may indicate conserved functions within the gene family, potentially facilitating rapid expression in response to biotic stress [35].

Plants, upon encountering environmental stressors, activate transcription factors that interact with specific cis-acting elements to regulate the expression of target genes, thus playing a vital role in their adaptive responses [43]. Within the *Pb-PR-1* gene family, a diverse array of cis-acting elements has been identified, including hormone-responsive motifs, light-responsive elements (such as the I box, TCCC-motif, and GATA-motif), and stress-responsive elements (notably MYB and MYC). The MeJA element stands out among the hormone response elements, acting as an activator of the jasmonic acid signaling pathway, which is critical in mediating plant defense responses and the synthesis of secondary metabolites [46]. In plants, MeJA response elements are intricately associated with the core transcription factor MYC2’s target site, G-box, as well as the W-box target site for the WRKY transcription factor, indicating a complex interplay in plant defense mechanisms and signal transduction pathways [20]. Among the Pb-PR-1 family, the proteins Pb-PR-1-3, Pb-PR-1-12, Pb-PR-1-18, Pb-PR-1-19, Pb-PR-1-25, and Pb-PR-1-28 exhibit notable structural deviations from other members, characterized by a reduced number of β-turns and a predominant composition of α-helices and unordered coils. This suggests that structural variations may be a key source of functional diversity among Pb-PR-1 proteins within cellular metabolism [47].

Defense enzymes are essential components of the plant immune system, with their activity closely linked to the antioxidant responses triggered by pathogen infection. The upregulation of these enzyme activities is crucial for neutralizing reactive ROS, thereby alleviating oxidative damage caused by pathogen invasion [48]. In this investigation, resistant cultivars exhibited elevated defense enzyme activities post-pathogen infection, demonstrating more significant changes compared to susceptible cultivars. Specifically, the activities of SOD and POD in resistant Duli began to decline on the fourth day post-inoculation, potentially reflecting the plant’s regulation of oxidative stress, reinforcement of cell walls, and suppression of pathogens [49]. Furthermore, increased CAT activity in resistant Duli may enhance the elimination of ROS caused by pathogen infection, providing additional cellular protection. Concurrently, the reduction in MDA content in resistant cultivars likely lessens oxidative damage to membrane lipids, thereby strengthening their disease resistance [22,50]. Previous studies have indicated that plants exhibiting high expression of the *MiPR1A* gene show enhanced activities of antioxidant enzymes (SOD, POD, and CAT), conferring increased resistance to infections by *C. gloeosporioides* [39]. The current study observed significant upregulation of the genes *Pb-PR-1-3*, *Pb-PR-1-7*, *Pb-PR-1-8*, *Pb-PR-1-9*, *Pb-PR-1-15*, *Pb-PR-1-16*, *Pb-PR-1-18*, *Pb-PR-1-26*, *Pb-PR-1-29*, and *Pb-PR-1-31* during the initial and mid-stages of infection (S2–S4), consistent with the trend observed in the early disease development where defense-related enzymes (SOD, POD, and PAL) are initially active before a subsequent decline. These findings suggest that these genes play significant roles in the early pathogenesis of fire blight in pears [51].

Genes located on the same branch of the phylogenetic tree or within the same chromosomal region exhibit marked variation in expression levels following pathogen infection. Notably, among those on the same branch, *Pb-PR-1-21*, and *Pb-PR-1-26* show a positive correlation with infection duration, whereas *Pb-PR-1-17* and *Pb-PR-1-19* demonstrate a negative correlation. Thus, the response of *PR-1* genes to fire blight induction appears unrelated to their evolutionary relationships or chromosomal positions, likely stemming from differences in their promoter cis-acting elements. Given that *PR-1* genes form a multigene family with substantial variability among members, their expression levels appear to increase at both transcriptional and translational levels in response to pathogen infection [52]. Upon pathogen infection, *PR-1* expression significantly rises, paralleling the mechanism by which host *PR* genes are upregulated, thereby enhancing the host’s defense capabilities [53]. Beyond their role in defense during pathogen infection, PR-1 proteins may also respond to abiotic stress stimuli [38]. This study indicates that members of the *Pb-PR-1* family may share similar functionalities in disease resistance. Additionally, investigating the interactions between these genes and other components of the plant’s immune system could provide a more comprehensive understanding of the resistance network. It would also be valuable to explore how these genes respond under different environmental conditions and pathogen strains, which could offer insights into breeding strategies for fire blight-resistant pear varieties.

## 4. Materials and Methods

### 4.1. Identification and Physicochemical Property Analysis of the PR-1 Gene Family

The genome assembly and gene annotation data for Duli were sourced from the BIG Data Genome Database at the Beijing Institute of Genomics, Chinese Academy of Sciences, under accession number GWHAAYT00000000. Reference sequences for *Arabidopsis* PR-1 proteins were retrieved from the Phytozome database (https://phytozome-next.jgi.doe.gov/, accessed on 20 September 2024). Local BLASTP alignments were executed within the Duli genome database with an *E*-value threshold of 1e–5. We extracted the gene IDs and CDS sequences for the PR-1 gene family members from the Duli genome. The extracted CDS is translated into protein using the Sequence Toolkit, ORF Prediction, and Batch Translate CDS to Protein. We uploaded the protein sequence to the NCBI Batch Web CD-Search Tool (https://www.ncbi.nlm.nih.gov/Structure/bwrpsb/bwrpsb.cgi, accessed on 20 September 2024), screened for the structural domains, and only kept proteins containing the CAP-PR-1 and CAPdomains with accession numbers CD05381 and CI00133. After screening, a total of 31 Pb-PR-1 genes were obtained. Protein sequences were analyzed for their physicochemical properties using EXPASY (http://web.expasy.org/compute_pi, accessed on 20 September 2024) [54]. Furthermore, the sub-cellular localization prediction for *Pb-PR-1* was performed utilizing WOLFPSORT (https://www.genscript.com/wolf-psort.html, accessed on 20 September 2024) [54].

### 4.2. Chromosomal Localization and Interspecific Collinearity Analysis of the Gene Family

In TBtools, we opened the “Graphics” tab, selected “Show Genes on Chromosome”, chose “Gene Location Visualize from GTF/GFF”, imported the GFF3 file of Duli and the ID of Pb-PR-1, entered the “Rename ID” and the “Chromosome Sort ID”, and then clicked “Start”. We retrieved the genome files of Arabidopsis (TAIR10_chr_all.fas) and gene annotation files (TAIR10_GFF3_genes) from the Arabidopsis Information Resource (TAIR) database. Inter-specific collinearity analysis was conducted using TBtools [55]. Adobe Illustrator 2022 was utilized to refine the imagery.

### 4.3. Constructing a Phylogenetic Tree for the Gene Family

Protein sequences of *PR-1* from closely related species were retrieved from NCBI. Firstly, the FASTA file was imported, and the meg format file was saved after sequence comparison. The meg file was then imported, and the “Construct/Test Neighbor-Joining Tree” option under PHYLOGENY was selected with the following parameter settings: bootstrap method with 1000 replicates, the VT+F+R4 model for nucleotide substitution, treatment of missing data by partial deletion, and a site coverage cutoff at 50%. The Interactive Tree Of Life (ITOL) online platform was utilized to enhance the visualization of the phylogenetic tree [56].

### 4.4. Conservation of PR-1 Protein Motifs, Gene Structure, and Protein Domain Analysis

We opened TBtools, clicked on “Others”, selected “MEME Suite Wrapper”, and chose “Simple MEME Wrapper”. We uploaded protein sequences from Pb-PR-1 genes, set the output path, and specified the number of motifs to find as 10, with a maximum E-value of 0.0001. We selected the mode for zero or one occurrence per sequence to obtain the MEME XML file. We used NCBI-CDD to acquire the conserved domain prediction results. Back in TBtools, we selected Gene Structure View, entered the protein ID, and uploaded the Duli GFF3 file, MEME XML file, and NCBI-CDD Excel sheet, then clicked Start to analyze and obtain the results.

### 4.5. Prediction of Cis-Acting Elements in the Gene Family

We opened TBtools, navigated to Graphics, selected Gene Structure View (Advanced), imported the Pb-PR-1 gene ID, GFF3 file, and ID rename file, and clicked Start. We used TBtools and the GTF/GFF3 Sequence Extract tool to extract the 2000 bp upstream of all coding sequences (CDS) of Duli. We also utilized Quick Fasta Extractor or Filter to extract the 2000 bp upstream of the Pb-PR-1 CDS. Next, we opened Fasta Sequence Manipulator to convert the sequence to uppercase. The extracted files were then submitted to the PlantCARE website [57] for predicting the cis-acting elements. The predicted table files were analyzed using Excel, imported into TBtools, and a schematic diagram of promoter cis-acting elements was drawn using Graphics and Simple BioSequence Viewer.

### 4.6. Prediction of Secondary Structure and 3D Modeling of Family Genes

We converted the protein sequences of the PR-1 gene family members to FASTA format. Then, we accessed the SOPMA tool via its online platform at SOPMA (NPS@: SOPMA secondary structure prediction—NPSA—Lyon—France). The protein sequences in FASTA format were pasted into the designated input area. We set the output width to 70. The other parameters were configured as follows: the number of conformation states was set to 4 (helix, sheet, turn, coil); the similarity threshold was set to 8; and the window width was adjusted to 17. After configuring these parameters, we submitted the sequences for analysis and performed graphical analysis based on the tool’s output. For the prediction and construction of the tertiary structure of proteins, SWISS-MODEL (https://swissmodel.expasy.org/, accessed on 20 September 2024) was employed [58].

### 4.7. Pathogen Activation

The *Erwinia amylovora* strain, originally preserved at the College of Horticulture, Xinjiang Agricultural University, and donated by Professor Hu Baishi from Nanjing Agricultural University, was retrieved from the −80 °C freezer and thawed on ice (strain number: NCPPB1665). Within a sterile work environment, a sterilized inoculation loop was used to gently touch the bacterial solution and streak it onto LB agar medium for activation at 28 °C for 48 h. Single colonies were selected and cultured in a constant temperature shaker at 28 °C and 180 rpm for 12–14 h until the optical density (OD 600) reached approximately 0.8, after which they were held in reserve.

### 4.8. In Vitro Leaf Inoculation and Enzyme Activity Assay

Fully expanded, disease-free shoots of Duli with tender leaves, both resistant and susceptible, were selected and washed. Incisions were made on both sides of the leaf midrib and perpendicular to it, followed by inoculation with an *Erwinia amylovora* bacterial solution. A total of 60 leaves were inoculated per treatment, with sterile water serving as a control. The leaves were subsequently placed in an artificial climate chamber set at 28 °C and 75% relative humidity for cultivation. The samples were collected from the peripheral area 1–2 cm away from the lesion region and stored in liquid nitrogen.

The determination of defense enzyme activity was conducted following the method described by Wang Beibei [50]. The nitro blue tetrazolium (NBT), guaiacol, catechol, and thiobarbituric acid (TBA) methods were employed to quantify the relevant defensive protective enzymes.

### 4.9. Real-Time Quantitative PCR (qRT-PCR)

Total RNA was extracted from both resistant and susceptible Duli leaves surrounding the lesion areas at 0 (control), 2, 4, and 6 days post-fire blight treatment, utilizing the RNAprep Pure Plant RNA Purification Kit (Tiangen, Beijing, China). Complementary DNA (cDNA) was synthesized via reverse transcription using the FastQuant cDNA First-Strand Kit (Kefute, Hangzhou, China) (Table 2 and Table 3).

Quantitative real-time PCR was conducted using the BlasTaq™ 2X qPCR MasterMix (Cat.NO:G891, abm, Richmond, Canada) kit. The qPCR reaction system was prepared according to Table 4. The real-time PCR program used was as follows: initial denaturation at 95 °C for 3 min, followed by 35 cycles of denaturation at 95 °C for 15 s, annealing at 56 °C for 20 s, and extension at 72 °C for 15 s. Each sample was amplified in triplicate. Specific primers were designed utilizing Primer3Plus (https://www.primer3plus.com/ accessed on 20 September 2024), with details provided in Supplementary Table S1, *YLS-8* (F: TGAGGTGCTGGCTTCTGT; R: TGACCGTTGATGGATCGTA) used to normalize expression levels of *Pb-PR-1* genes. Relative expression levels were determined employing the 2^−ΔΔ^Ct method.

### 4.10. Data Analysis

Data from the experiments were organized using Microsoft Excel 2016. A single-factor analysis of variance (ANOVA) was conducted on the measured indicators using SPSS version 23, with a significance level set at *p* < 0.05. Data visualization was performed using Prism version 9.

## 5. Conclusions

In this study, we identified 31 members of the *Pb-PR-1* gene family, which can be classified into four distinct subgroups, and all members belong to the CAP superfamily. Following infection with fire blight, we observed a significant increase in the activities of PPO and CAT in resistant Duli, demonstrating highly significant differences when compared to susceptible strains. qRT-PCR analysis further revealed that the expression levels of the majority of *Pb-PR-1* family genes in resistant Duli were elevated post-infection relative to their susceptible counterparts. Notably, the expression levels of *Pb-PR-1-11*, *Pb-PR-1-21*, and *Pb-PR-1-26* exhibited a direct correlation with the duration of the infection. These findings suggest that these genes may play a pivotal role in the resistance responses of Duli to fire blight.

## Figures and Tables

**Table 1:** Physiological properties and subcellular localization of the *Pb-PR-1* gene family.

Sequence ID	Number of Amino Acid (aa)	Molecular Weight (Da)	pI	Instability Index	Aliphatic Index	Grand Average of Hydropathicity	Subcellular Localization
Pb-PR-1-1	194	22,221.31	9.85	34.55	57.84	−0.632	Vacuole.
Pb-PR-1-2	341	37,556.87	8.81	38.33	59.59	−0.870	Nucleus.
Pb-PR-1-3	340	37,544.85	8.94	38.97	58.91	−0.882	Vacuole.
Pb-PR-1-4	151	16,709.98	9.85	43.45	66.49	−0.505	Vacuole.
Pb-PR-1-5	173	18,781.16	8.97	36.61	75.03	−0.245	Vacuole.
Pb-PR-1-6	196	22,468.42	5.80	27.56	62.76	−0.382	Vacuole.
Pb-PR-1-7	186	21,503.33	5.39	27.69	61.40	−0.403	Vacuole.
Pb-PR-1-8	200	22,804.20	9.63	42.76	68.75	−0.481	Vacuole.
Pb-PR-1-9	170	19,296.70	9.25	42.81	60.35	−0.455	Vacuole.
Pb-PR-1-10	146	15,282.81	4.67	43.08	61.58	−0.400	Vacuole.
Pb-PR-1-11	140	14,606.29	4.77	38.64	76.07	−0.159	Vacuole.
Pb-PR-1-12	163	17,199.18	4.86	43.50	71.90	−0.266	Vacuole.
Pb-PR-1-13	160	17,836.25	6.09	41.40	63.44	−0.416	Vacuole.
Pb-PR-1-14	160	18,203.49	4.96	38.14	69.56	−0.438	Vacuole.
Pb-PR-1-15	160	17,504.76	5.51	38.31	70.19	−0.142	Vacuole.
Pb-PR-1-16	160	17,835.12	5.90	38.04	73.19	−0.254	Vacuole.
Pb-PR-1-17	166	18,534.98	6.88	32.79	67.53	−0.395	Vacuole.
Pb-PR-1-18	168	18,805.92	5.67	21.85	62.74	−0.426	Vacuole.
Pb-PR-1-19	168	18,722.90	6.04	22.06	63.27	−0.460	Vacuole.
Pb-PR-1-20	168	18,706.85	6.04	21.89	60.95	−0.492	Vacuole.
Pb-PR-1-21	123	13,268.70	9.69	25.16	60.24	−0.518	Vacuole.
Pb-PR-1-22	127	13,543.99	7.69	36.17	73.86	−0.335	Vacuole.
Pb-PR-1-23	160	17,200.27	8.69	37.09	73.19	−0.284	Vacuole.
Pb-PR-1-24	178	19,785.51	9.35	41.97	71.80	−0.354	Vacuole.
Pb-PR-1-25	240	26,547.70	5.74	37.02	82.88	−0.365	Nucleus.
Pb-PR-1-26	161	17,697.60	5.64	24.10	69.07	−0.478	Vacuole.
Pb-PR-1-27	154	16,491.48	6.01	27.30	79.16	−0.084	Vacuole.
Pb-PR-1-28	151	16,123.67	5.34	23.89	57.48	−0.444	Vacuole.
Pb-PR-1-29	161	17,169.90	5.10	28.27	63.60	−0.293	Vacuole.
Pb-PR-1-30	161	17,079.88	5.10	31.37	69.07	−0.227	Vacuole.
Pb-PR-1-31	161	17,089.90	5.10	29.50	71.49	−0.230	Vacuole.

**Table 2:** cDNA removal reaction system.

Reagent	Volume (μL)
5×gDNA Buffer	2 μL
Total RNA	6 μL
RNase-Free ddH_2_O	2 μL
5×gDNA Buffer	2 μL

**Table 3:** Reverse transcription reaction system.

Reagent	Volume (μL)
10×Fast RT Buffer	2 μL
RT Enzyme Mix	6 μL
FQ-RT Primer Mix	2 μL
RNase-Free ddH_2_O	2 μL

Incubated at 42 °C for 15 min and then at 95 °C for 3 min to obtain the cDNA product, which was immediately stored at −20 °C for later use.

**Table 4:** Real-time PCR reaction system.

Component	Volume (μL)
BlasTaq^TM^ 2×qPCR MM	10 μL
Forward Primer	0.4 μL
Reverse Primer	0.4 μL
Template DNA	1.5 μL
Nuclease-free H_2_O	7.7 μL

## Data Availability

Data are contained within the article.

## Supplementary Materials

The following supporting information can be downloaded at https://www.mdpi.com/article/10.3390/ijms26115074/s1.

## Abbreviations

The following abbreviations are used in this manuscript:

SOD	Superoxide dismutase
POD	Peroxidase dismutase
PPO	Polyphenol oxidase
CAT	Catalas
PAL	Phenylalanine ammonia-lyase
MDA	Malondialdehyde
DL	Duli
RDL	Resistant Duli
