# Supplementary material for: Genome-Wide Identification of the PR-1 Gene Family in Pyrus betulaefolia Bunge and Its Expression Analysis Under Fire Blight Stress
Source: Int J Mol Sci. 2025 May 24;26(11):5074. doi: 10.3390/ijms26115074 (PMC12155532; doi:10.3390/ijms26115074)

Supplementary data：

Supplementary Table S1. qRT-PCR specific primers

Primer name	Primer sequence（5'-3'）
Pb-PR-1-1F	TCACAACACTCAGAGATCAAGG
Pb-PR-1-1R	CGAATGAACCAAGGAGCAGT
Pb-PR-1-2F	TCATCACGACTGTCAAATGC
Pb-PR-1-2R	GTACACCTTCTCATGTCC
Pb-PR-1-3F	ACCATAATACTCACCATCTC
Pb-PR-1-3R	CCTTGACTGCCTTGCCAATG
Pb-PR-1-4F	GCAGGCTAGTGAGGTACC
Pb-PR-1-4R	CCACTGTAGTTGTAGAA
Pb-PR-1-5F	AGTACCTCCAAGCTCACAACC
Pb-PR-1-5R	TACTTGCTGCCACTGGTG
Pb-PR-1-6F	ATCCTCTTTGCTCTCTACC
Pb-PR-1-6R	CCACTTTGCTGCCCTCA
Pb-PR-1-7F	ATCCTCTTTGCTCTCTACC
Pb-PR-1-7R	CTCTCCTATGCAACCCCAGC
Pb-PR-1-8F	TACACTCGACGGGGCCTTACG
Pb-PR-1-8R	TGTCCTTCCACACCATTTGC
Pb-PR-1-9F	AAGCCAGGCCAACCAATTTG
Pb-PR-1-9R	TGCGTACCATTGTGCATACC
Pb-PR-1-10F	AAGCATTCGGGTACAGG
Pb-PR-1-10R	TGAGTCACGCCATACCACCT
Pb-PR-1-11F	TGTGAACTTAAGCATTCC
Pb-PR-1-11R	CGTTGGTGCTGTAATCATAATC
Pb-PR-1-12F	CAGAGAGCGCCAAAAACTATGC
Pb-PR-1-12R	ATAAGTCACCGTCAGGGCTTAC
Pb-PR-1-13F	GAATGTGGTCACTACCTTGCTG
Pb-PR-1-13R	ATGGGATCGTAGCTGCAAAC
Pb-PR-1-14F	CTTCATCAGGGAACACAAC
Pb-PR-1-14R	GCTCCATTTGGCAGTCCTCG
Pb-PR-1-15F	CATCTGCTCCGTGGCATTAATC
Pb-PR-1-15R	TTGATCGGACAGACACCAACC
Pb-PR-1-16F	CCGATCAAATGGAACGAGACC
Pb-PR-1-16R	ATACTTGACAGCAGCTGCAC
Pb-PR-1-17F	ACCCCACCAAGACTTCATAGAC
Pb-PR-1-17R	GCGAATGCACCATTTCACAC
Pb-PR-1-18F	CGTCGATGAACACAACAGAGC
Pb-PR-1-18R	ACCCTCTCACACTTGTATTGGC
Pb-PR-1-19F	ACTTGGCTGAAGGCTATGGAG
Pb-PR-1-19R	TCCGCATTCATCACCAACAC
Pb-PR-1-20F	CGTCGATGAACACAACAGAGC
Pb-PR-1-20R	ATGTCACACCCTCTCACTCTTG
Pb-PR-1-21F	CGAGAATTGGTGACTGC

Pb-PR-1-21R	TTAGAGTTGTAATCGTAG
Pb-PR-1-22F	CAGCCGTGAACTTGTTTGTG
Pb-PR-1-22R	ATTTACGGCCTGATGATGCC
Pb-PR-1-23F	CAGCCGTGAACTTGTTTGTGAG
Pb-PR-1-23R	ATTGTTGCACCGCACTTTCG
Pb-PR-1-24F	ATGTAGGGTGCGCAAAAGTG
Pb-PR-1-24R	TGCCACTTTCTGCACCAATG
Pb-PR-1-25F	AGGCTCAACCCTAATCCAATCG
Pb-PR-1-25R	TGGTTGGCGTGATTTTGTGC
Pb-PR-1-26F	ACCCCAAGACTTCCTCAATTCC
Pb-PR-1-26R	TTGGTTGGCGTGGTTTTGTG
Pb-PR-1-27F	TGCACAAAAGTACGCCAACC
Pb-PR-1-27R	TCCCAACATGTCACTAGTGCTC
Pb-PR-1-28F	AAACTACGCCAACCAACACG
Pb-PR-1-28R	CCGTTCCTGACATGTCAACAC
Pb-PR-1-29F	GAGTCGAACTCTTGTGCTGATG
Pb-PR-1-29R	ACTGCTGCACCTCACTTTTG
Pb-PR-1-30F	AAACTACGCCAACCAACACG
Pb-PR-1-30R	CCGTTCCTGACATGTCAACAC
Pb-PR-1-31F	ACATGTTGGCGACTGCAATC
Pb-PR-1-31R	TCAGCACACGAGTTCGACTC
YLS-8-F	TGAGGTGCTGGCTTCTGT
YLS-8-R	TGACCGTTGATGGATCGTA

Supplementary Table S2. Protein secondary structure analysis

Gene Name	Percentage %				Distribution of secondary structure elements
	alpha-helix	β-folding	curl up irregularly	extended chain	
Pb-PR-1-1	25.26	4.12	13.92	56.70	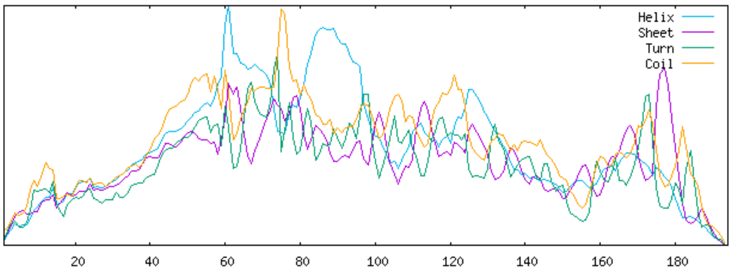
Pb-PR-1-2	16.72	1.47	5.87	75.95	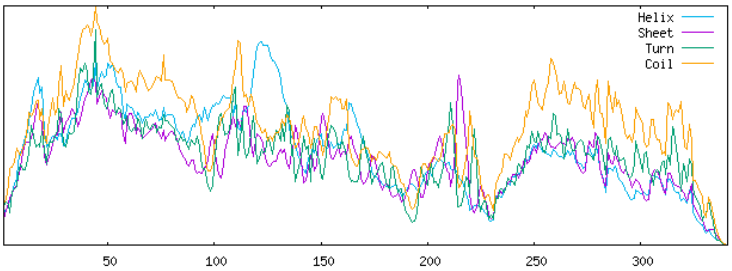
Pb-PR-1-3	13.53	1.76	8.82	75.88	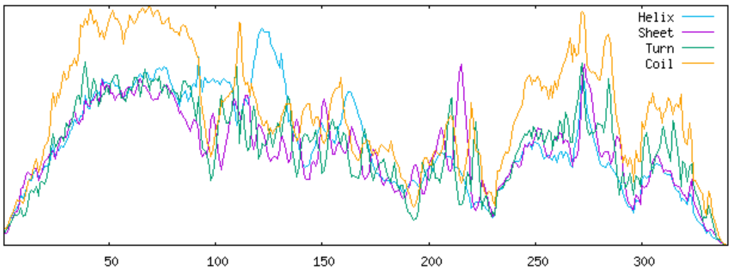
Pb-PR-1-4	27.15	2.65	15.89	54.30	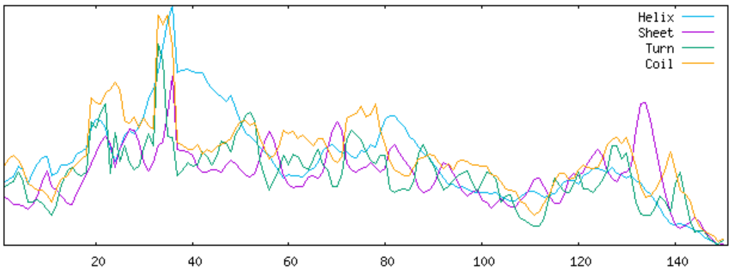
Pb-PR-1-5	33.53	3.47	13.29	49.71	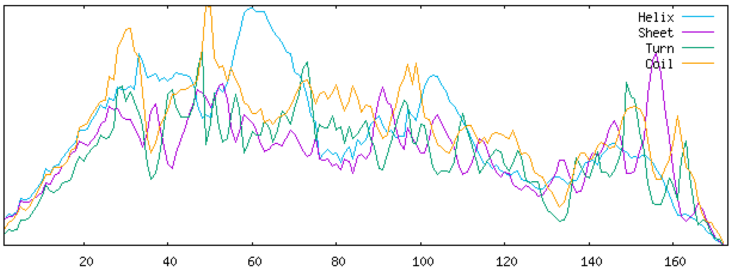

Pb-PR-1-6	22.96	2.55	14.29	60.20	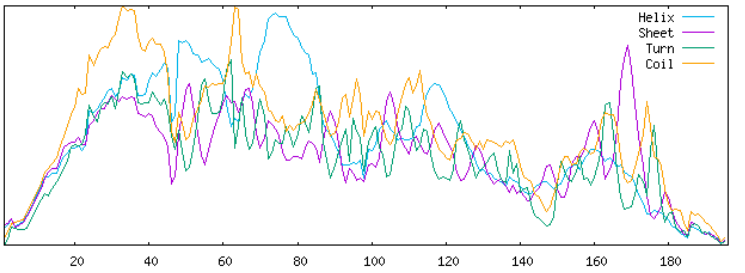
Pb-PR-1-7	27.96	3.23	14.52	54.30	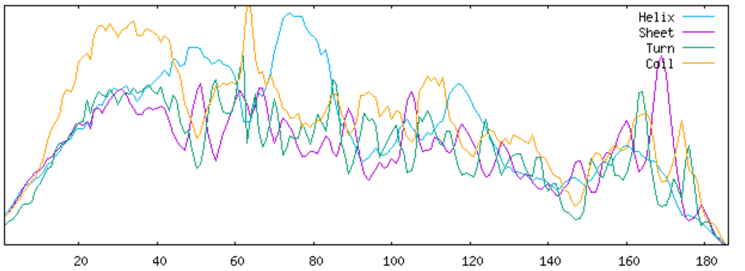
Pb-PR-1-8	31.50	3.00	12.50	53.00	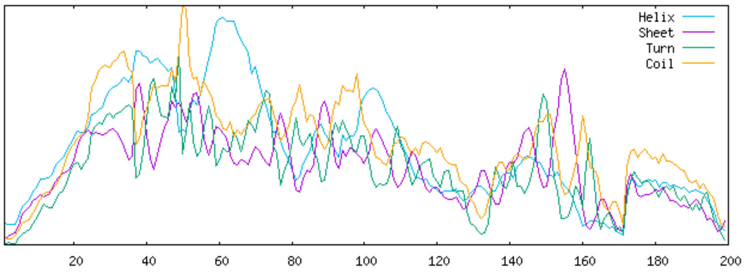
Pb-PR-1-9	38.24	4.12	13.53	44.12	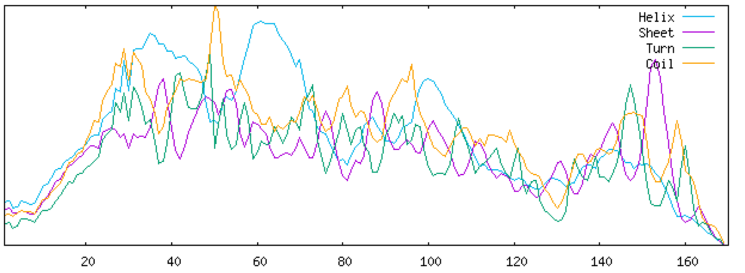
Pb-PR-1-10	29.45	5.48	15.75	49.32	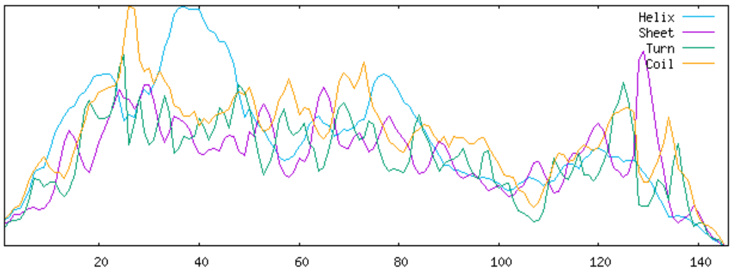
Pb-PR-1-11	27.86	2.14	15.71	54.29	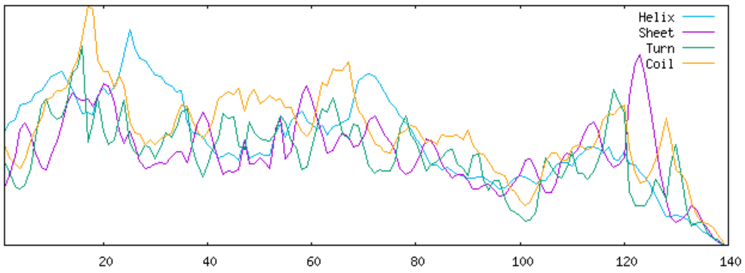

Pb-PR-1-12	36.81	4.29	14.72	44.17	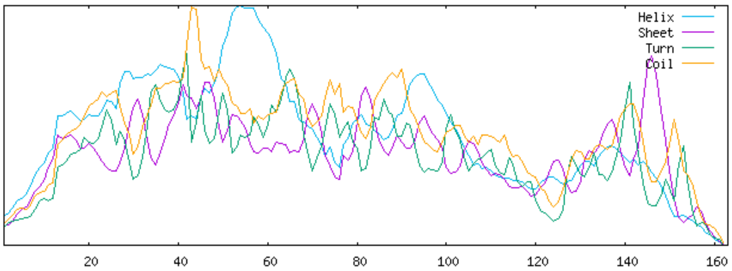
Pb-PR-1-13	35.62	4.38	13.12	46.88	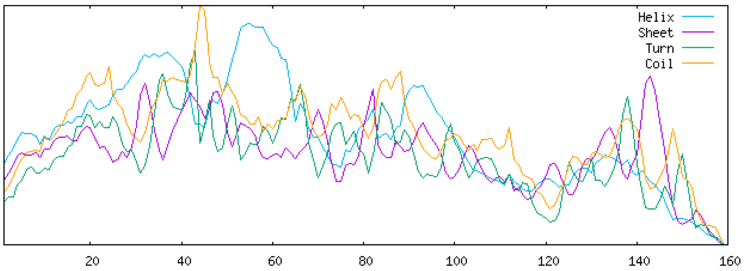
Pb-PR-1-14	36.88	4.38	10.62	48.12	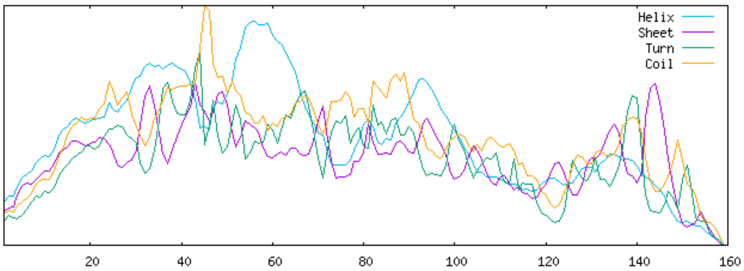
Pb-PR-1-15	38.12	6.25	13.12	42.50	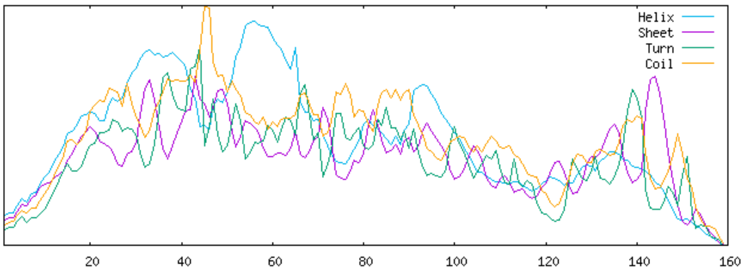
Pb-PR-1-16	39.38	3.75	11.25	45.62	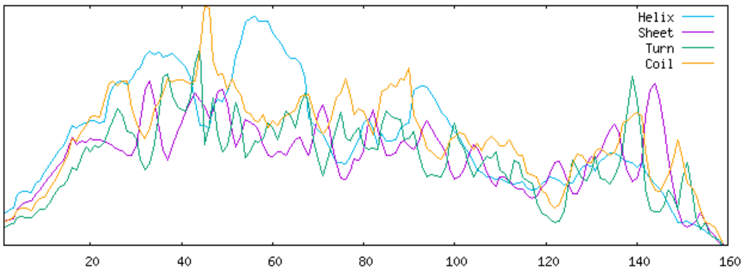
Pb-PR-1-17	37.35	3.61	14.46	44.58	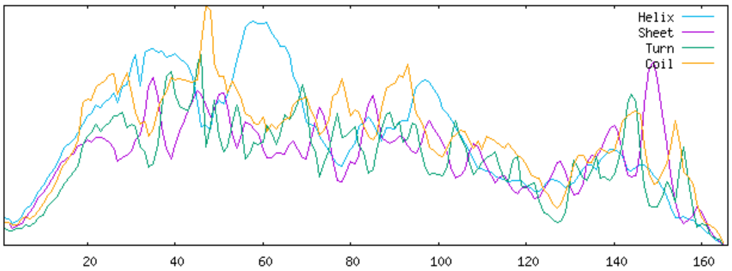

Pb-PR-1-18	32.14	4.17	13.69	50.00

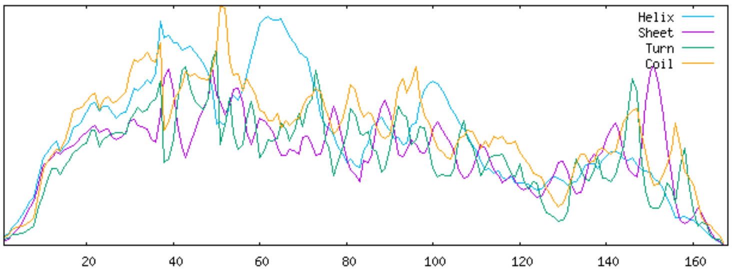

Pb-PR-1-19	30.36	4.17	13.10	52.38

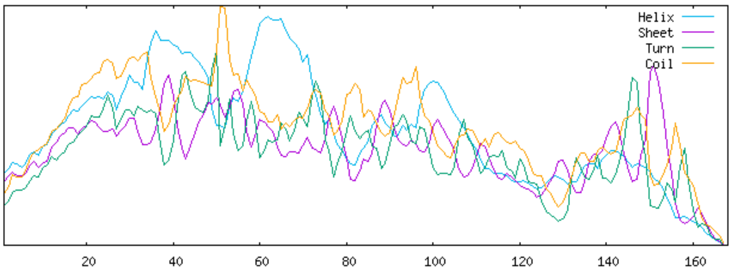

Pb-PR-1-20	35.71	4.17	13.10	47.02

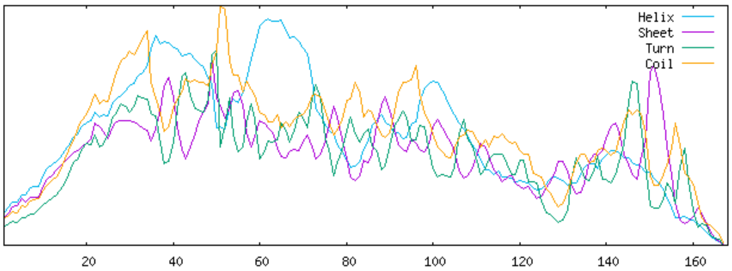

Pb-PR-1-21	34.96	1.63	6.50	56.91

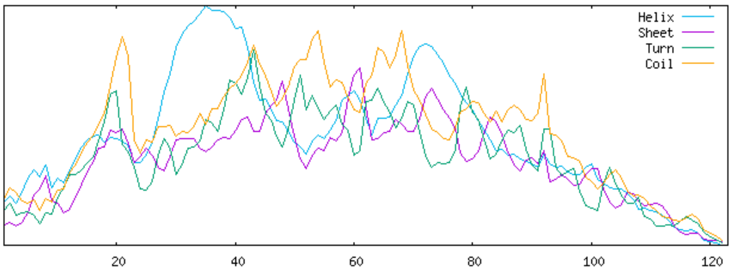

Pb-PR-1-22	44.88	3.15	8.66	43.31

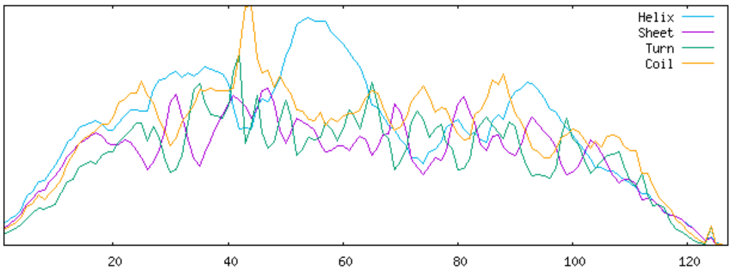

Pb-PR-1-23	36.88	5.62	15.00	42.50

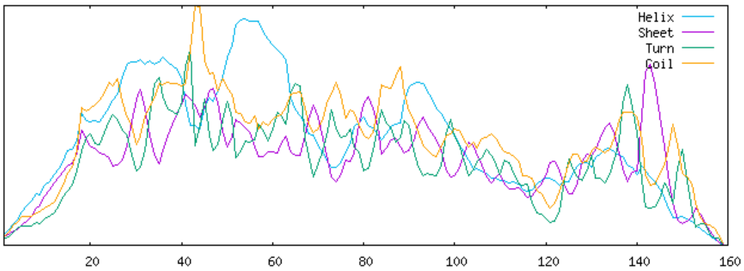

Pb-PR-1-24	23.03	4.49	12.92	59.55	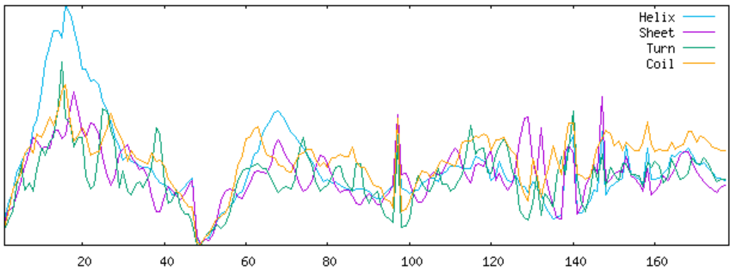
Pb-PR-1-25	30.42	4.17	9.58	55.83	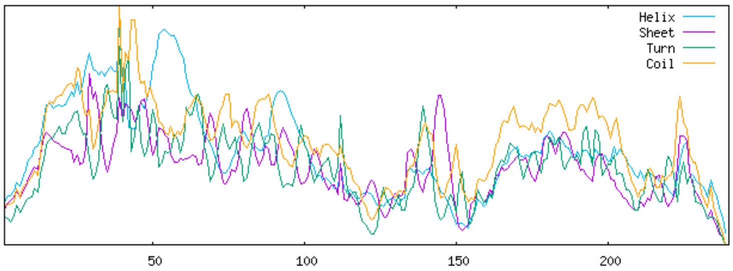
Pb-PR-1-26	37.89	3.11	14.91	44.10	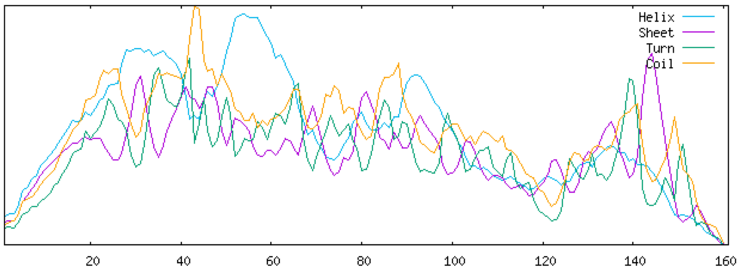
Pb-PR-1-27	37.66	2.60	15.58	44.16	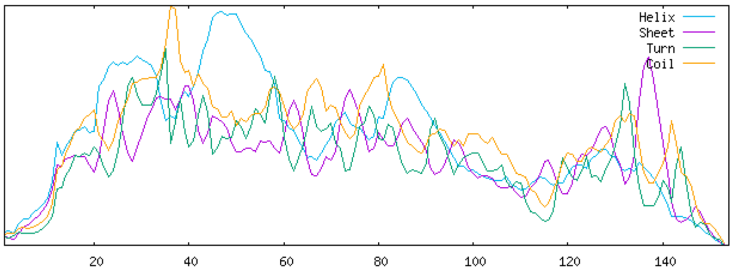
Pb-PR-1-28	32.45	3.97	15.89	47.68	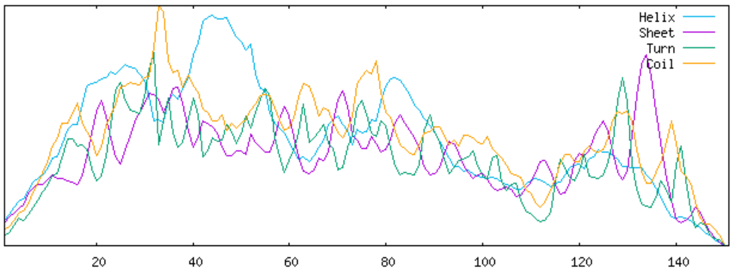
Pb-PR-1-29	36.02	3.73	14.91	45.34	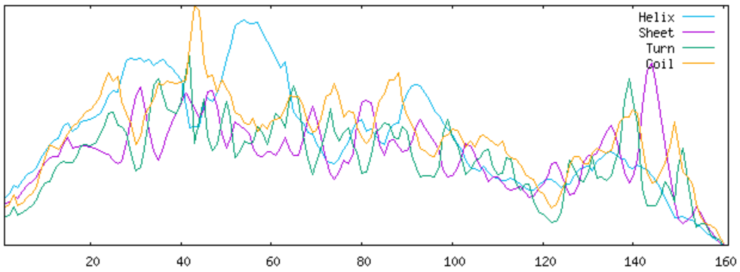

Pb-PR-1-30	36.02	3.73	14.91	45.34	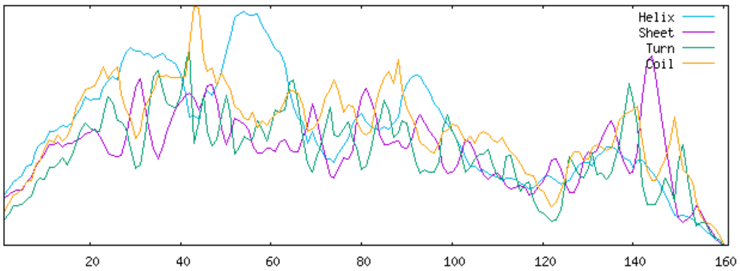
Pb-PR-1-31	35.40	3.73	14.91	45.96	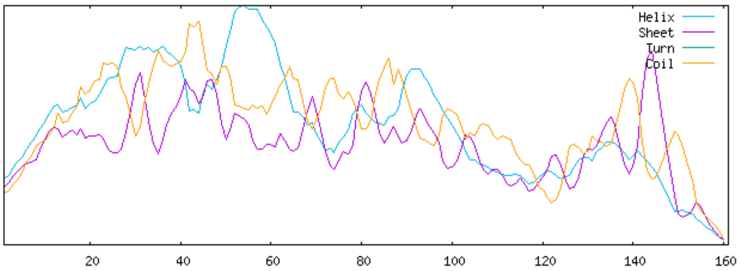

Relative gene expression results is not the same for all pictures.

Supplementary Figure S1. Relative expression of the Pb-PR-1 gene after inoculation with fire blight

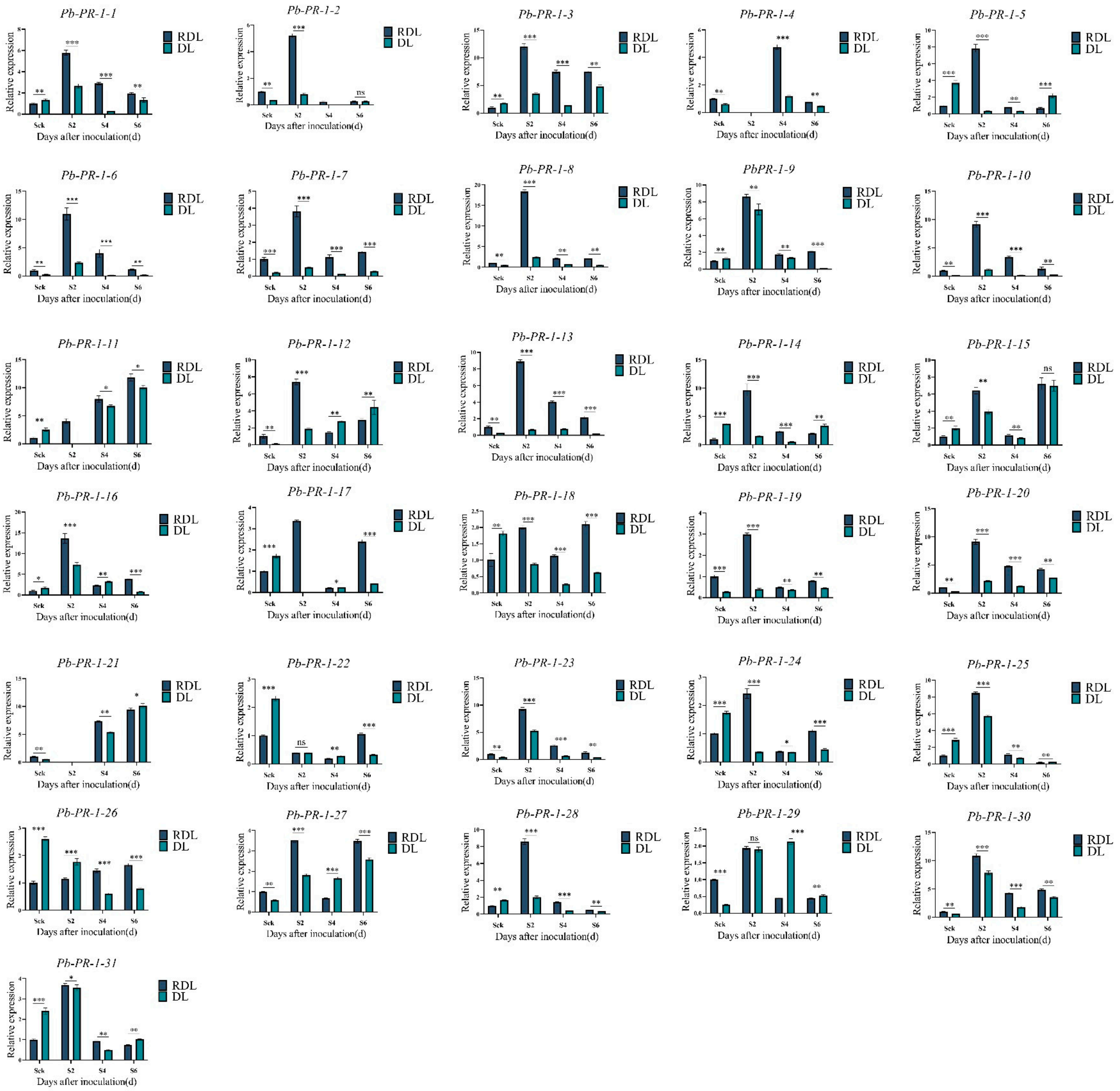

Supplement: Supplementary file 1 [file ijms-26-05074-s001.zip › ijms-3598026-supplementary.pdf]
